# Generation of knockout rabbits with X-linked severe combined immunodeficiency (X-SCID) using CRISPR/Cas9

**DOI:** 10.1038/s41598-020-66780-6

**Published:** 2020-06-19

**Authors:** Yoshiko Hashikawa, Ryuhei Hayashi, Masaru Tajima, Toru Okubo, Shohei Azuma, Mitsuru Kuwamura, Naofumi Takai, Yasuyuki Osada, Yayoi Kunihiro, Tomoji Mashimo, Kohji Nishida

**Affiliations:** 1 Department of Ophthalmology, Osaka University Graduate School of Medicine, Osaka, Japan; 2 Institute of Large Laboratory Animal Sciences, Osaka University Graduate School of Medicine, Osaka, Japan; 3 Department of Stem Cells and Applied Medicine, Osaka University Graduate School of Medicine, Osaka, Japan; 4 Institute of Experimental Animal Science, Graduate School of Medicine, Osaka University, Osaka, Japan; 5 Osaka Prefecture University School of Life and Environmental Sciences Veterinary Pathology, Osaka, Japan; 6 KITAYAMA LABES CO.,LTD., Nagano, Japan; 7 Laboratory Animal Research Center, Institute of Medical Science, The University of Tokyo, Tokyo, Japan; 8 Integrated Frontier Research for Medical Science Division, Institute for Open and Transdisciplinary Research Initiatives (OTRI), Osaka University, Osaka, Japan

**Keywords:** Allotransplantation, Immunological models

## Abstract

Severe immunodeficient mice are widely used to examine human and animal cells behaviour *in vivo*. However, mice are short-lived and small in size; while large animals require specific large-scale equipment. Rabbits are also commonly employed as experimental models and are larger than mice or rats, easy to handle, and suitable for long-term observational and pre-clinical studies. Herein, we sought to develop and maintain stable strains of rabbits with X-linked severe combined immunodeficiency (X-SCID) via the CRISPR/Cas9 system targeting *Il2rg*. Consequently, X-SCID rabbits presented immunodeficient phenotypes including the loss of T and B cells and hypoplasia of the thymus. Further, these rabbits exhibited a higher success rate with engraftments upon allogeneic transplantation of skin tissue than did wild type controls. X-SCID rabbits could be stably maintained for a minimum of four generations. These results indicate that X-SCID rabbits are effective animals for use in a non-rodent model of severe immunodeficiency.

## Introduction

Immunodeficient animals are important for *in vivo* transplantation studies using human or animal cells. In particular, immunodeficient mice are most often used because of their high reproductive ability and ease of handling. Nude mice with a *Foxn1* mutation have been widely used in studies on cancer as athymic mice^1,2^. Thereafter, SCID mice with *Prkdc* mutations were discovered^3^, which succeeded in differentiating human haematopoietic stem cells in the mouse body upon transplantation of human foetal liver and thymus tissues under the renal capsule; these mice are referred to as SCID-hu and are widely used in studies on HIV^4^. *Il2rg*-mutant mice have recently been developed through gene targeting technology^5^; this gene is essential for signal transduction among interleukins such as IL-2, IL-4, IL-7, IL-9, and IL-15.

*Il2rg* is located on the X chromosome, and a mutation of the γ chain in humans causes X-SCID^6^. Examples of X-SCID-animals include rats^7^, marmosets^8^, pigs^9^, and dogs with the gene mutation^10^. Furthermore, functional defects such as T, B, and/or NK loss lead to severe immunodeficiency.

Although numerous mouse models of immunodeficiency have been reported and handling of animals is relatively easy, they are small in size and short-lived, thus complicating surgical procedures in certain regions including the eye and blood vessels and resulting in inadequate long-term *in vivo* evaluation. In contrast, large animals such as pigs and monkeys can have a longer lifespan and larger organ size; however, they require advanced handling expertise and a large-scale facility, resulting in a higher cost. Moreover, large animals have a long period of sexual maturity and gestation period.

Rabbits can be operated on relatively easily owing to their size. Furthermore, rabbits are the second most widely used animal after experimental rodent models owing to their long life, ease of handling, and the ability to be reared in a small space. Further, they have a short period of sexual maturity and gestation and are more genetically related to humans than other rodents^11^. Hence, rabbits are regularly employed in studies of infectious disease^12^, liver cancer^13^, adult T cell leukaemia-lymphoma^14^, joint or bone disease^15,16^, dentistry^17^, eye disease (cornea^18^, retina^19^), cardiovascular disease^20,21^, atherosclerosis^22–24^, and lipid metabolism^25^ and for the production of antibodies^26^. The detailed rabbit genome has recently been published in the Broad Institute server (https://www.broadinstitute.org/rabbit/rabbit-genome-project) and RGB-Net: Collaborative European Network on Rabbit Genome Biology (http://www.biocomp.unibo.it/rabbit/index.php). Numerous studies have reported rabbits as models of human disease through genetic modification^27–29^.

Although several rabbit models of immunodeficiency^30,31^ have been developed, no study has functionally analysed the lineage stability of *Il2rg* gene-deficient rabbits. This study reports the development of *Il2rg*-KO rabbits and maintenance of a stable strain, using the CRISPR/Cas9 system.

## Results

### Injection of *Il2rg* encoding CRISPR/Cas9 mRNA into rabbit embryos

**Figure 1:**
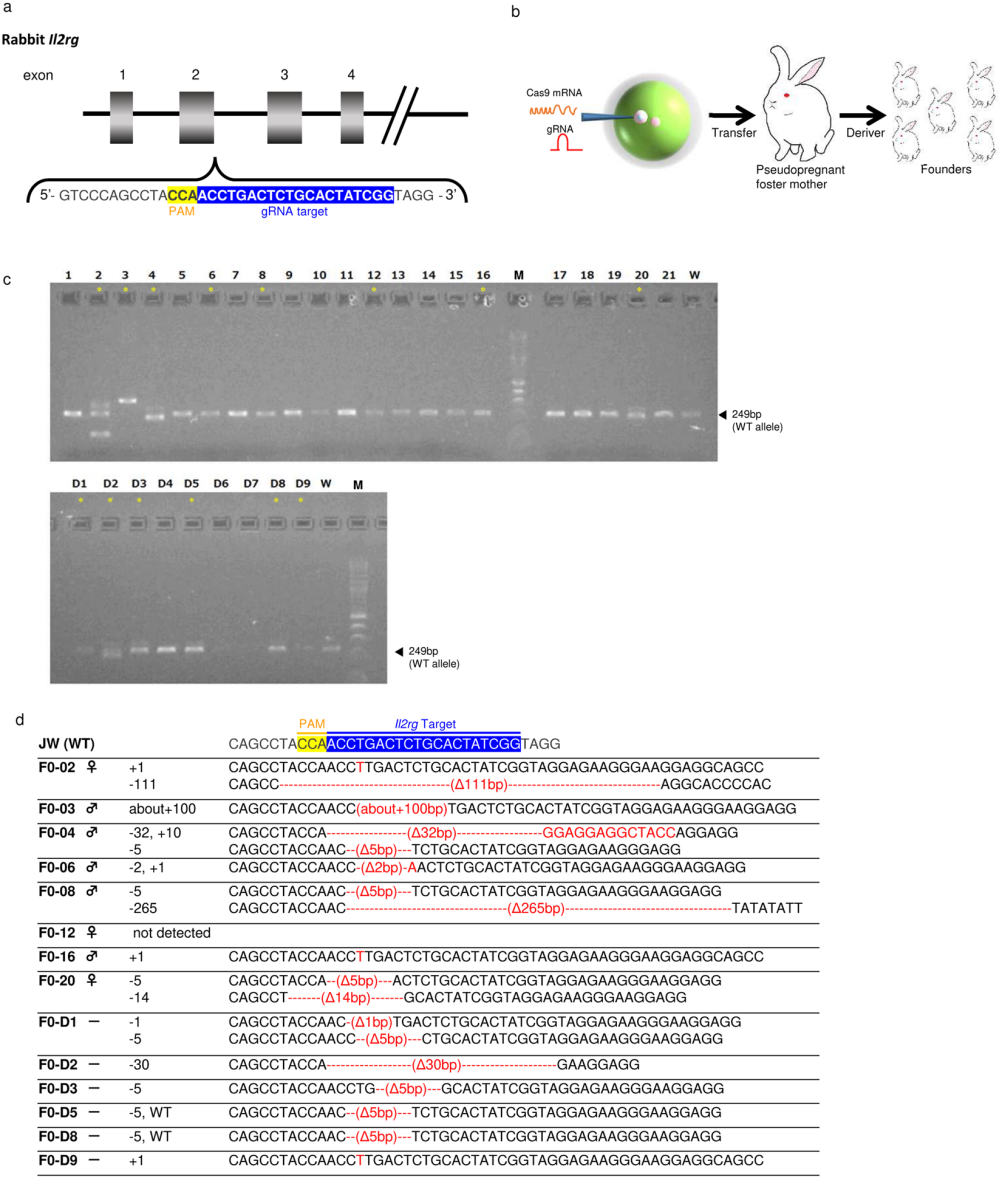
Verification of founder *Il2rg*-mutant rabbits using the CRISPR/Cas9 system. **(a)** Schematic representation of rabbit *Il2rg*. Magnified views illustrate the binding sites for CRISPR. **(b)** Co-injection of gRNA targeting rabbit *Il2rg* with Cas9 mRNA into rabbit male pronuclei embryos resulted mutations at the target sites. **(c)** PCR analysis of 30 offspring obtained through CRISPR/Cas9 injection of JW oocytes. Fourteen mutants (#2, 3, 4, 6, 8, 12, 16, 20, D1, D2, D3, D5, D8, and D9) were identified (asterisk). The product size of the wild-type JW Rabbit allele (arrow) is of 249 bp. **(d)** Sequencing analysis of CRISPR/Cas9-induced mutations in the target region in JW rabbits. Multiple deletions and insertions are depicted by red dashes and letters, respectively, and are aligned along the WT sequences displayed on the top line.

Cas9 and gRNA concentrations were divided into two conditions: low and high concentrations (Table 1). At low concentration conditions, 293 embryos were harvested from ten donor rabbits, of which 241 survived (82.3%). Twenty-two (9.1%) offspring from founder animals were obtained, of which eight (36.3%) were mutated. At high concentration conditions, 259 embryos were harvested from ten donor rabbits, of which 194 survived (74.9%). Eight (4.1%) offspring were obtained from founder animals, of which 6 (75.0%) were mutated. Sequencing analysis of the CRISPR/Cas9 target site in these 14 founder animals revealed that the offspring harboured various mutations including 1–265-bp deletions and a 1–about 100-bp insertion in the region overlapping the CRISPR/Cas9 target site (Fig. 1c,d).

**Table 1:** Generation of *Il2rg*-mutated rabbits with the CRISPR/Cas9 system.

Cas9 RNA (ng/μL)	guide RNA (ng/μL)	No. Oocyte injected	No. (%) Transferred embryo	No. (%) Pups born	No. (%) Mutants
100	50	293	241 (82.3)	22 (9.1)	8 (36.3)
200	100	259	194 (74.9)	8 (4.1)	6 (75)
total		552	435 (78.8)	30 (6.8)	14 (46.7)

Three of the five male offspring harboured mosaicism biallelic mutations at the *Il2rg* locus despite having only one X chromosome, suggesting that mosaicism was induced upon CRISPR/Cas9 treatment, a situation frequently observed in the DNA of transgenic founders. Two of the three female offspring harboured biallelic mutations. We maintained the strain with the 265-bp deletion as it was easy to identify gene disruption and numerous amino acid deletions were expected.

### Germline transmission of *Il2rg*-deficient X-SCID rabbits

Hemizygous male (*Il2rg−/Y*) rabbits display the severe immunodeficiency phenotype (X-SCID); however, heterozygous female (*Il2rg−/+*) rabbits display a normal phenotype^9,32^. Therefore, hemizygous male (F0-8, *Il2rg−/ Y*) and WT female rabbits were mated to produce heterozygous female (*Il2rg−/+*) offspring with a 265-bp deletion in the F1 generation (Fig. S1). Furthermore, in the F2 generation, 21 of 80 (26.3%) hemizygous male rabbits were obtained and analysed (Table S1). Moreover, 6 of 22 (27.3%) hemizygous male rabbits among the F3 offspring and 8 of 42 (19.0%) hemizygous male rabbits among the F4 offspring were obtained (Table S1).

### Characterisation of *Il2rg*-deficient X-SCID rabbits

**Figure 2:**
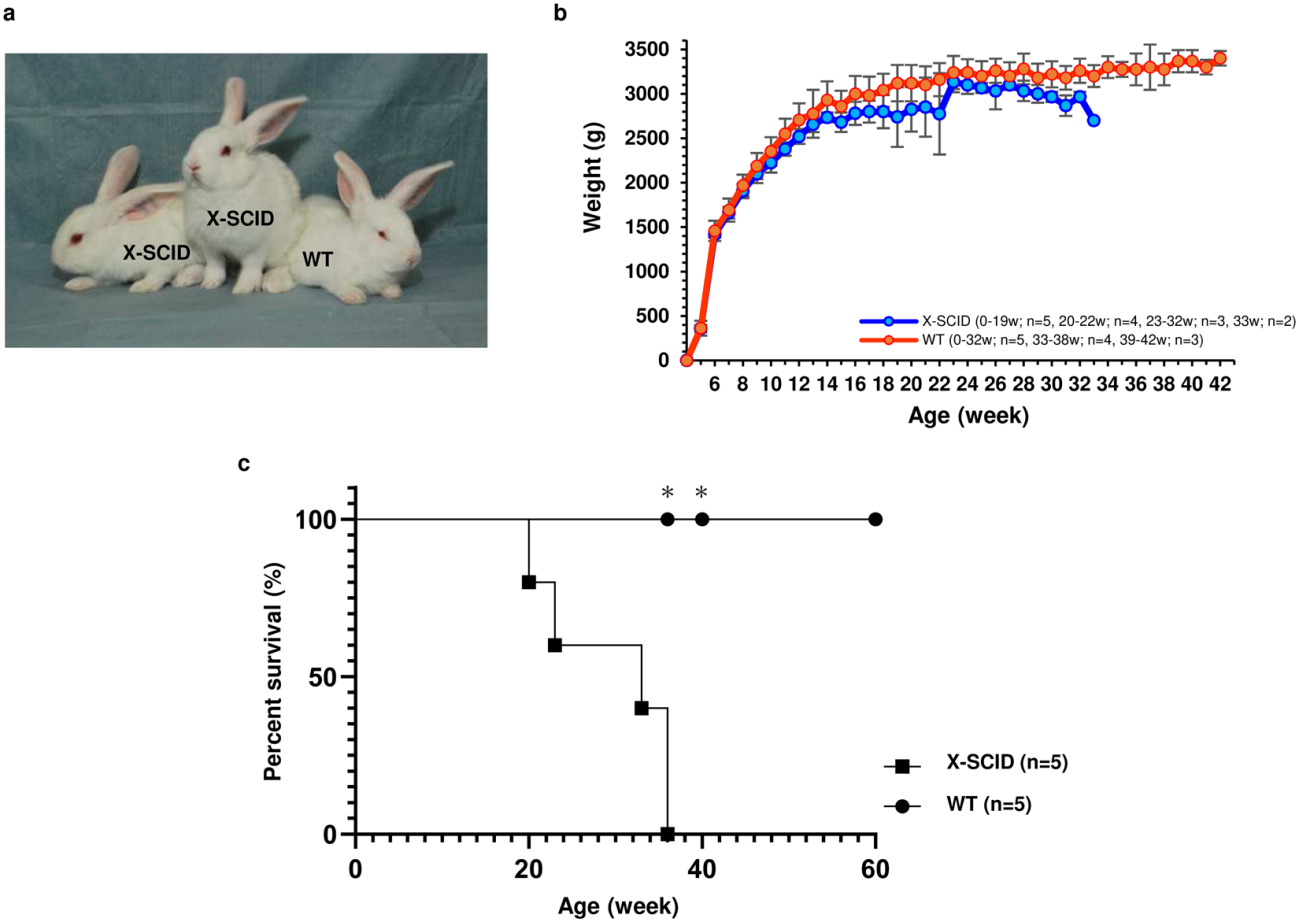
Postnatal growth and survival curve of X-SCID and WT rabbits. **(a)** Photograph of four-week-old male X-SCID (*Il2rg−/Y*) and WT (*Il2rg* + */Y*) rabbits. **(b)** Postnatal growth of rabbits. X-SCID (0–19w; n = 5, 20–22w; n = 4, 23–32w; n = 3, 33w; n = 2), WT (0–32w; n = 5, 33–38w; n = 4, 39–42w; n = 3). **(c)** Survival curve of rabbits. X-SCID n = 5, WT n = 5. * Two of the five animals were euthanized at 38 and 40 weeks for another experiment.

Appearances did not markedly differ between X-SCID and WT rabbits (Fig. 2a). The body weight of male WT rabbits tended to increase until approximately 14 weeks of age, followed by a gradual increase. Furthermore, the body weight of X-SCID rabbits tended to increase until approximately 14 weeks; however, thereafter, individuals with a decreased body weight were noted (Fig. 2b). Survival rates were confirmed among five X-SCID rabbits and five WT in F2 offspring. The mean lifespan of X-SCID rabbits was 29.6 ± 6.8 weeks. Four of the five X-SCID rabbits were euthanised owing to lung abnormalities (black discoloration of lung tissue (Fig. S2)), etc., and the remaining rabbits were experimentally euthanised. Two of five WT animals were experimentally euthanised, while the remaining three were alive during the assessment period (Fig. 2c).

Haematological parameters determined from peripheral blood (PB) profiles, including the white blood cell (WBC) count, were lower than those in WT rabbits (Table 2), and differential leukocyte counts revealed a significant reduction in lymphocytes in the X-SCID rabbits.

**Table 2:** Differential leukocyte counts of 20-week-old male X-SCID (*Il2rg−/Y*) and WT (*Il2rg* +*/Y*) rabbits p-value *P < 0.01, **P < 0.001.

	WBC*(×10^3^/µL)	Differential WBC count (%)					
		Bas	Eos	Neut**	Lym**	Mon	Other
X-SCID (*Il2rg-*/*Y*) n=4	1.8 ± 0.5	6.9 ± 0.8	5.0 ± 1.4	76.9 ± 4.3	8.2 ± 3.2	2.4 ± 0.7	0.8 ± 0.3
WT (*Il2rg+*/*Y*) n=4	4.9 ± 0.4	4.4 ± 0.7	2.6 ± 0.9	14.9 ± 3.6	77.5 ± 3.3	0.6 ± 0.1	0.1 ± 0.0

**Figure 3:**
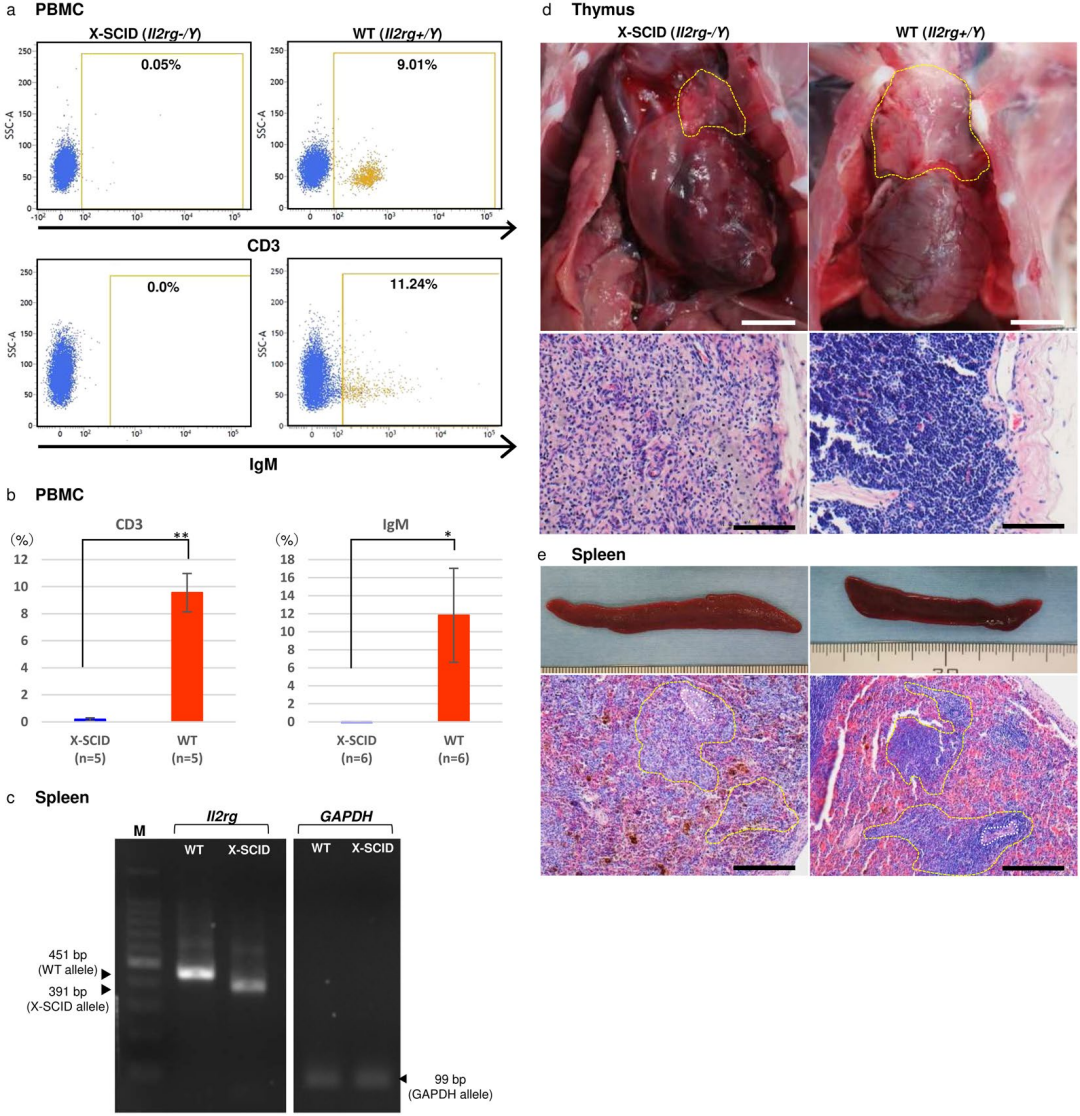
Phenotype of peripheral lymphocytes and thymic and spleen organogenesis in X-SCID rabbits. **(a)** Flow cytometric analysis of cell populations isolated from peripheral blood lymphocytes. Dot plots for CD3 and IgM for the differentiation of T- and B-cell sub-populations. The numbers shown are mean percentages. *P < 0.05, **P < 0.001. **(b)** Flow cytometric analysis of cell populations isolated from among peripheral blood lymphocytes. **(c)** RT-PCR analysis of *Il2rg* in the spleen in control X-SCID and WT rabbits. *Gapdh* was considered the internal control. **(d)** Histological analysis of H&E-stained thymus specimens (yellow circled area) of X-SCID (20× magnification) and WT (20× magnification) rabbits (36 weeks old, male). The thymus of X-SCID rabbits was markedly hypoplastic. Scale bar, 100 μm. **(e)** Histological analysis of H&E-stained spleen specimens of X-SCID (10× magnification) and WT (10× magnification) rabbits (36 weeks old, male). The yellow circled area indicates white pulp and the white circled area indicates the central artery. In the X-SCID spleen, the white pulp was devoid of lymphocytes. Scale bar, 100 μm.

Flow cytometry analysis of cell populations isolated from peripheral blood mononuclear cells revealed a marked reduction in the number of lymphocytes in X-SCID rabbits (Fig. 3a,b). The number of CD3 + T cells (X-SCID, 0.2% ± 0.1%; WT 9.6% ± 1.4%; n = 5 each, p < 0.01) and IgM+ B cells (X-SCID, −0.05% ± 0.06%; WT 11.8% ± 5.2%; n = 5 each, p < 0.05) were significantly decreased in X-SCID rabbits. Heterozygous female rabbits exhibited normal lymphoid development and were indistinguishable from WT rabbits.

RT-PCR analysis revealed a reduction in mRNA size in the spleen of X-SCID rabbits in comparison with WT rabbits (Fig. 3c).

Gross and microscopic analyses revealed that X-SCID rabbits underwent abnormal lymphoid development. In the thymus, it was difficult to isolate the exact thymus tissue from X-SCID rabbits for measuring the weights because of the remarkable thymus hypoplasia. Instead, we examined thymus tissue by HE-staining, indicating that thymus from X-SCID rabbits contained almost no lymphocytes (Fig. 3d). Although spleen weight and appearance did not markedly differ, lymphocytes in the white pulp area were reduced (Fig. 3e, Table S2). The mesenteric lymph node and Peyer’s patches in the ileum were not macroscopically identified. The appendix wall in X-SCID rabbits was thinner than that in WT rabbits (Fig. S3a–c).

### Allogenic skin transplantation

**Figure 4:**
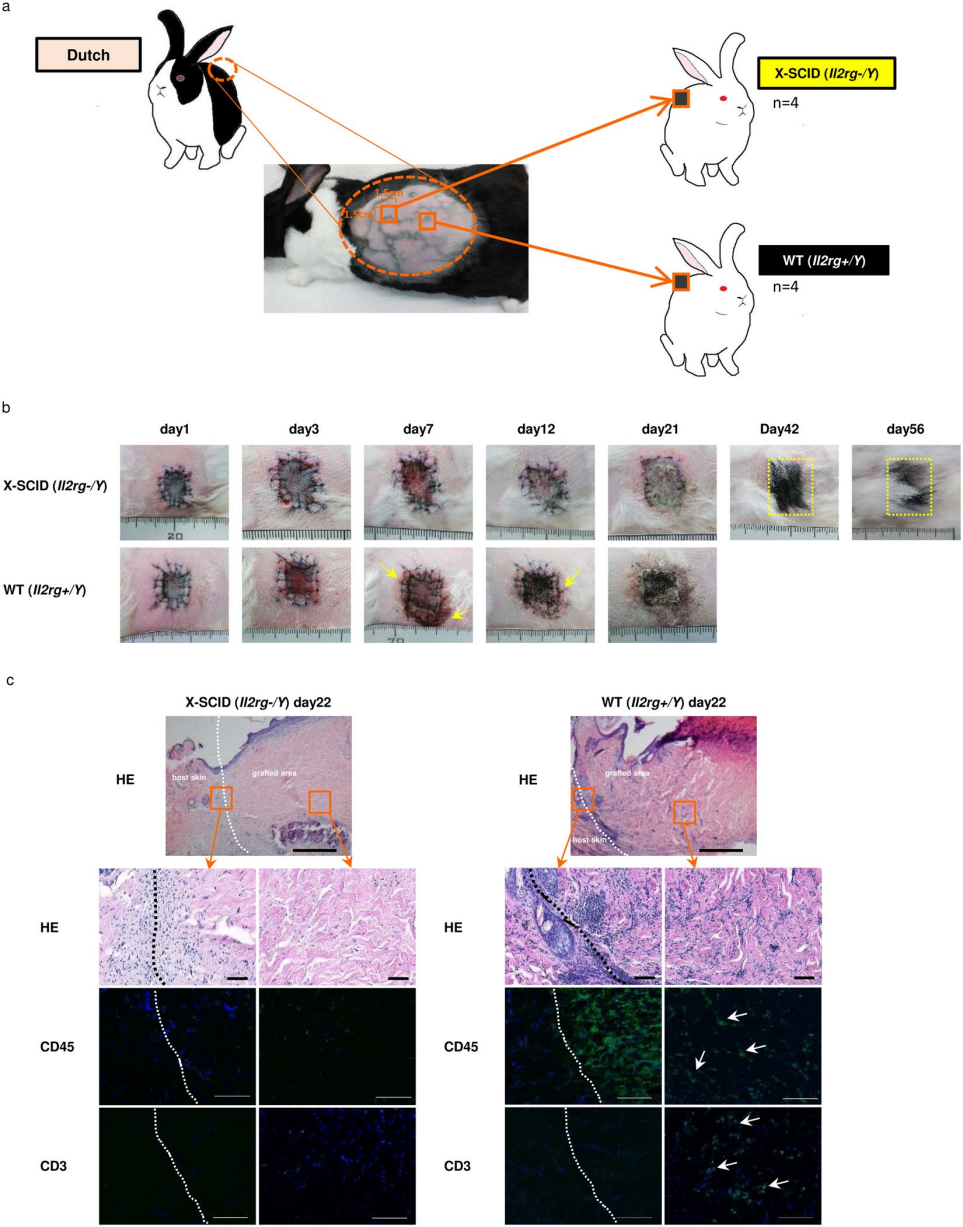
Allogeneic transplantation of skin. **(a)** Skin graft method. **(b)** Morphological observation of the skin graft of time-dependent change. Yellow circled area is black hair of Dutch rabbit. **(c)** Histological analysis of the day 22 skin graft section of X-SCID and WT rabbits stained with H&E (X4), CD45 (X200), CD3 (X200). Scale bar 500μm. White dot lines are the boundary of the skin graft and original skin. Lymphocytes are infiltrating at the border of WT, but lymphocyte infiltration is scarcely shown in X-SCID.

For skin transplantation, Dutch rabbits (21–22 weeks old, n = 2) were used as donors and X-SCID rabbits (21–22 weeks old, n = 4, F2 offspring) and WT rabbits (21–22 weeks old, n = 4, F2 offspring) were used as recipients (Fig. 4a). No marked difference was observed until three days after transplantation. On day 7, although the exudate was observed in WT rabbits, no clear difference was observed. After 12 days, the skin graft hardened and darkened in all WT rabbits, yet remained soft state in normal skin colour in X-SCID rabbits. On day 21, the skin graft remained dark colour and was not found to aggulutinate in WT, however, remained normal skin colour and aggulutinated in X-SCID rabbits. After 56 days, black hair was observed in X-SCID rabbits, which is characteristic of Dutch rabbit (Figs. 4b, S4a,b).

Histological examination via haematoxylin and eosin staining in X-SCID rabbits revealed marked fibroblast proliferation at the interface between the transplanted and original skin, and no inflammatory cell infiltration, including lymphocytes, was observed. However, in WT rabbits, numerous lymphocytes were observed at the interface (Fig. 4c). During immunostaining, while few positive cells were observed in X-SCID rabbits (Fig. 4c). However, in WT rabbits, CD45-positive cells were confirmed at the border between the site of transplantation and the autologous skin, and CD3-positive cells were confirmed in the dermis (Fig. 4c).

## Discussion

This study reports the development of established X-SCID rabbits via the CRISPR/Cas9 system and their stable maintenance, demonstrating that *Il2rg*-KO rabbits can be generated with a high genome editing efficiency of 46.7%. The efficiency was relatively high compared to that of *Il2rg*-KO model mice^5^ (31%; ES cells), and rats^7^, (24%; ZFN), rabbits^31^, (16.7%; CRISPR/Cas9), pigs^9^, (1.25%; somatic cell clone), and marmosets^8^, i.e., (42.6%; TALEN and ZFN). In a previously generated rabbit model of immunodeficiency^30^, the genome-editing efficiency was 100%; however, all founder rabbits died in 45 days. Our X-SCID rabbit model and the previously generated rabbit model of immunodeficiency^30,31^ were subjected to the same *Il2rg* gene KO; however, the target sites were different, thus also being associated with genome editing efficiency and lineage stability. Non-rodent experimental animals including rabbits are often used for long-term follow-up evaluation studies such as transplantation experiments. Rabbits yield offspring over a short period, with 4–5 months of sexual maturity and a gestational age of approximately 30 days in comparison with other large animals (e.g., cynomolgus monkeys with a 3.5–4-year period of sexual maturity and a gestational period of 150–225 days)^33^. The ability to obtain highly efficient progeny (F1/F2) that can rectify mosaic mutations and epigenetic defects in the 6th month in the shortest period leads to shortening of the study period, which is advantageous for research and development^34^.

The *Il2rg−/Y* (X-SCID) rabbits herein presented marked hypoplasia of the thymus, a decreased lymphocyte count in the white pulp area in the spleen, loss of peripheral lymph nodes, and a significant reduction in the lymphocyte count in peripheral blood, similar to other X-SCID animals. These observations indicate that in rabbits as well, the *Il2rg* plays contributes to the development and differentiation of lymphocytes and its functional deficiency induces immunodeficiency symptoms. X-SCID rabbits displayed a significant reduction in the number of CD3+ T cells among peripheral blood mononuclear cells during sexual maturation. In X-SCID mice and marmosets, T cells increase in infancy ^8,35^. In human X-SCID, CD3+ T cells are almost completely obliterated^6^; hence, rabbit phenotypes can be considered similar to those of humans. Furthermore, the number of B cells remains constant in WT pigs and human X-SCID^6,9^; however, the number of B cells is significantly reduced in mice and rats^5,7^. Herein, the number of IgM+ B cells also significantly reduced in the rabbits, displaying the same tendency as that of mice and rats. NK cells were not detected as no specific antibody to rabbit NK cells was identified, as previously reported^31^. T/B-cell fractions differ among animal species with age. Herein, T and B cells were significantly reduced during sexual maturation of rabbits; however, in X-SCID rabbits, further detailed subset analysis of T and B cells in each immune organ and further analyses are required to elucidate the development and differentiation of T and B cells.

Furthermore, we performed allogeneic transplantation and confirmed skin engraftment among individuals with genetically different characteristics. Skin transplantation is a well-established method often used in studies on the immune response and rejection^36,37^, and skin transplantation is a non-invasive method. In our skin transplantation experiments, we used Dutch rabbits as donors and Japanese White rabbits (X-SCID rabbits) as recipients. Dutch rabbits have black hair, while X-SCID rabbits have white hair; hence, it is easy to ascertain graft acceptance. The Dutch rabbits belong to the Dutch germline *Oryctolagus cuniculus*; while the Japanese White species belong to Japanese germline *O. cuniculus*. The genomes of Dutch and Japanese White rabbits belong to different groups^38^. Although the innate immune system was functional in the X-SCID rabbits, we were able to confirm the engraftment of allogeneic skin in these rabbits. T and B cells are largely involved in the rejection of transplanted immunity and it has been shown that both the number and function of T and B cells are decreased in X-SCID rabbits. The degree of rejection varies depending on the type of organ (tissue), and among them, skin tissue is susceptible to rejection^39^. Therefore, survival can potentially be achieved in the transplantation of organs (tissues) other than the skin. Furthermore, in X-SCID pigs^9^, lymphoid cell reconstruction was confirmed via allogeneic bone marrow transplantation, suggesting that X-SCID rabbits with the same phenotype can be expected to be used as a model for immune system reconstruction through bone marrow transplantation.

We also investigated whether X-SCID rabbits can stably maintain their lineages. Consequently, we obtained a stable line of rabbits up to the F4 generation. The average lifespan of the F2 generation X-SCID rabbits was approximately 200 days, and the X-SCID pigs survive for a longer period than the average period of approximately 70 days^9^, probably owing to antibiotic administration and housing conditions. Antibiotics were used from 6 weeks after weaning, and the previous studies on X-SCID rats^41^ have reported that the infection was suppressed through antibiotic administration. Furthermore, in a previous study, rabbits were maintained under conventional housing conditions^30^, in contrast, our rabbits were maintained in a clean booth until they were approximately 9–15 weeks old. Furthermore, inbreeding degeneration is rapid among rabbits^40^; however, the issue was resolved through mating between a heterozygous individual with the same phenotype as that of WT and a WT fertile individuals raised in another colony. From the F2 generation, pneumonia symptoms and diarrhoea, which are considered to be caused by *Pneumocystis oryctollagi*, were observed. This condition has also been reported in X-SCID rats and other immunodeficient rabbits^41,42^. A previous study reported^42^ that before weaning, X-SCID rabbits were resistant to *Pneumocystis* owing to immunity acquired from the mother; however, severe infection was observed sometimes after weaning. These results suggest that the lung abnormality did not result from gene knockout itself but rather from *Pneumocystis* pneumonia due to a suppressed immune response after weaning. Furthermore, WT rabbits harbouring an antibiotic specific for *Pneumocystis* pneumonia have reduced *P. oryctollagi* and the individuals are used as parent WT rabbits, and the offspring are obtained through caesarean sections and reared under aseptic conditions, resulting in the suppression of *Pneumocystis* infections in the same manner as X-SCID rats^43–45^. Thus, we speculate that X-SCID rabbits survive longer and efficient strain maintenance is possible.

We thus generated highly immunodeficient animals. X-SCID rats were confirmed with regards to human ovarian cancer cells and survival of human embryonic cells or human induced pluripotent stem cell (iPSC)-derived DA neurons^46^. X-SCID rabbits with the same phenotype can be expected to be used as humanised animal models transplanted with human-type organs (tissues) or pluripotent stem cells. Furthermore, the number of transplantable cells is limited because *Il2rg*-KO mice and X-SCID rats have a small body size. X-SCID rabbits can be transplanted with a similar or equal number of cells to that of humans. Safety tests including those related to regenerative medicine and cell therapy are potentially applicable because they are considered advantageous for optimising the dosage of transplanted cells and for confirming the characteristics of grafted cells at the transplant site. Accordingly, rabbits are potentially applicable as a link between mice and rats and other animal species including pigs and monkeys, and as an excellent tool for studies on organ and cell transplantation, cancer, and haematopoietic system bridging to humans.

## Methods

### Animals

Japanese White (JW) rabbits were obtained from KITAYAMA LABES Japan (Nagano, Japan) and housed under conditions of 30–70% humidity and a 12:12-h light/dark cycle. They were fed with pellets which had been sterilised via gamma irradiation (50 kGy/box) and supplied with water treated using a reverse osmosis system (Edstrom Japan, Co., LTD., Saitama, Japan). All X-SCID rabbits were subcutaneously injected twice per day with 10 mg/kg enrofloxacin from six weeks of age.

All animal care and experimental protocols conformed to the Guidelines for Animal Experiments of Osaka University and were approved by the Animal Experimental Committee of Osaka University. All of the experiments using recombinant DNA were approved by the Recombinant DNA Committees of Osaka University and were performed in accordance with our institutional guidelines.

### Construction of the CRISPR/Cas9 vector and gRNA design

A Cas9 expression plasmid (RDB13130) was used herein. To generate a Cas9 mRNA expression vector, plasmids were linearized with Nhe1 (New England Biolabs, Ipswich, MA, USA), and extracted with NucleoSpin Gel and PCR Clean-up kits (Macherey-Nagel, Duren, Germany) in accordance with the manufacturer’s instructions. After purification of the linearized DNA, Cas9 mRNA was transcribed *in vitro* using a mMESSAGE mMACHINE T7 Ultra Kit (Thermo Fisher Scientific, Waltham, MA, USA) and was purified using a MEGAClear kit (Thermo Fisher Scientific) and resuspended in RNase-free water, in accordance with the manufacturer’s instructions.

gRNA was designed using CRISPR DESIGN software (http:crispr.mit.edu/). gRNAs were transcribed *in vitro* using a MEGAshortscript T7 Transcription Kit (Thermo Fisher Scientific) in accordance with the manufacturer’s instructions from synthetic double-stranded DNA containing a T7 promoter, 20-bp target sequences, and gRNA tail sequences (Integrated DNA Technologies, San Diego, CA, USA). The gRNA DNA template was synthesised *in vitro* using CUGAR 7 *in vitro* Transcription Kit (NIPPON GENE, Tokyo, Japan) in accordance with the manufacturer’s instructions and transcribed. mRNA was purified using a MEGAClear kit (Thermo Fisher Scientific) in accordance with the manufacturer’s instructions and resuspended in RNase-free water^47,48^.

### Embryo microinjection and transfer

Sexually matured JW female rabbits (n = 39, KITAYAMA LABES), as donors, were super-ovulated via subcutaneous injection of follicle-stimulating hormone (FSH, Antrin R10, Kyoritsu Seiyaku, Tokyo, Japan) six times per day. Seventy-two hours after FSH injection, the donor females were artificially inseminated, and a single dose of 75 IU human chorionic gonadotropin (hCG, GONATROPIN1000, ASKA Pharmaceutical, Tokyo, Japan) was immediately intramuscularly administered to induce ovulation. Simultaneously, 75 IU of hCG was injected to sexually mature recipient female rabbits to induce pseudopregnancy. Nineteen to 21 h after hCG injection, fertilised eggs were harvested from donor females and pronuclear-stage embryos were selected and cultured in a modified medium (Medium199 [1×] Hanks‘ Salts [Thermo Fisher Scientific]) supplemented with 5% FBS (Biological Industries, Cromwell, CT, USA) before the microinjections. Using a micromanipulator (Narishige, Tokyo, Japan), 200 ng/uL Cas9 mRNA, 100 ng/ul gRNA, 15 ng/ul pExo1 or 100 ng/ul Cas9 mRNA, 50 ng/ul gRNA, 15 ng/ul pExo1 were microinjected into the male pronuclei of embryos. These embryos were cultured in a modified medium (Medium199 [1×] Earle’s Salts; Thermo Fisher Scientific) supplemented with 20% FBS (Biological Industries) after the microinjections. Surviving embryos were sorted and twenty to thirty embryos were transplanted into the oviduct of recipient rabbits. A part of the tail of few-day old pups tail was sampled and genome DNA was extracted using the KAPA Express Extract Kit (Roche Sequencing and Life Science Kapa Biosystems, Wilmingto, MA, USA). PCR was performed using the following conditions: 94 °C for 1 min, 35 cycles of 98 °C for 10 s, 61 °C for 15 s, and 68 °C for 30 s, and final extension at 72 °C for 1 min (Table S3). The PCR products were then directly sequenced using the BigDye Terminator v3.1 cycle sequence mix and Applied Biosystems 3130 DNA Sequencer (Thermo Fisher Scientific) standard protocol.

### Physiological characteristics

Body weights of rabbits (F2 offspring X-SCID [n = 5] and WT [n = 5]) were assessed once a week. Rabbits were monitored throughout the observation period and mortalities were recorded daily (F2 offspring X-SCID [n = 5] and WT [n = 5]). Blood parameters were analysed in 20-week-old, X-SCID (n = 4) and control WT (n = 4) F2 offspring. Approximately 600 μL peripheral blood was sampled from the ear vein. Blood parameters including the blood cell count and a differential WBC count were measured using ADVIA2120 (Siemens Healthineers Headquarters, Erlangen, Germany).

### Fluorescence activated cell sorting (FACS) analysis of T and B lymphocytes

Flow cytometric analyses were performed for 17–25-week-old, *Il2rg-/Y* (n = 4–5) and control WT (n = 5–6) F2 offspring. To enumerate T and B lymphocytes in whole blood, approximately 2 mL of peripheral blood was sampled from the ear vein in a heparin-coated syringe. Lymphocytes from blood were separated using a Ficoll-Paque PREMIUM (GE Healthcare UK Ltd., Bucks, UK) gradient via centrifugation for 40 min at 400 × *g*. Cells were filtered with a 40-μm cell strainer (BD Biosciences, San Diego, CA, USA) and washed with PBS to eliminate debris. The remaining pellets were resuspended in 1 ml of PBS and enumerated with a haemocytometer. Anti-human CD3 (F7.2.38, Dako Denmark A/S, Glostrup, Denmark), anti-rabbit IgM (MCA812GA, Bio-Rad, Hercules, CA, USA) were used as primary antibodies. Mouse IgG1 antibodies (Biolegend, San Diego, CA, USA) were used as isotype-matched controls. For intracellular protein staining, a BD Cytofix/Cytoperm (BD Biosciences) kit was used in accordance with the manufacturer’s instructions.

Approximately 2.5–5.0 × 10^5^ cells per sample were incubated with the primary antibodies in PBS for 30 min on ice and washed twice via centrifugation at 400 × *g* for 5 min. Thereafter, cells were incubated with Alexa Fluor 647-conjugated secondary antibodies (1:200; Thermo Fisher Scientific) under the same conditions. After washing, the cells were resuspended with 500 μL PBS before FACS analysis. Thereafter, 10,000 live cells were used for flow cytometry analysis using FACSVerse (BD Biosciences). The data were analysed using the BD FACSDiva Software (BD Biosciences).

### RT-PCR analysis

Total RNA was extracted from the spleen of 36-week-old F2 offspring using QIAzol reagent (Qiagen, Valencia, CA, USA). Reverse transcription was performed using the SuperScript III First-Strand Synthesis System for RT-PCR (Thermo Fisher Scientific) in accordance with the manufacturer’s protocol, and cDNA was used as a template for PCR. The cycling conditions were as follows: 98 °C for 10 s, followed by 40 cycles at 60 °C for 15 s and 68 °C for 30 s (Table S3). PCR products were analysed via electrophoresis on 2.0% agarose gels. *Gapdh* was used as an internal control refer to previous study^49^.

### Allogenic skin transplantation

The rabbits were anaesthetised via isoflurane inhalation 4–5% and maintained at 2–3%. The dorsae of the rabbits were shaved, and the surgical area was disinfected with Povidone-Iodine and washed with 70% ethanol using sterile cotton, and finally dried with sterile gauze. The surgical procedure was carried out under aseptic conditions.

The skin graft donor site was located on the dorsa and a 1.5 × 1.5-cm^2^ area was marked and harvested using scissors and a scalpel. Immediately thereafter, the grafts stored in HBSS (Thermo Fisher Scientific) at 4 °C until grafting. The recipient rabbits were inflicted with a full-thickness excision skin wound of 1.5 × 1.5 cm^2^ on the dorsa, using a scalpel and scissors. The skin graft was grafted at the dorsal wound in recipient rabbits, using 5–0 PROLENE (Johnson & Johnson, New Brunswick, NJ, USA) sutures, with one stitch at each of four corners and 3–4 stitches on each side. The skin graft sites were covered with a transparent dressing (Tegaderm, 3 M, St. Paul, MN, USA), and sterile gauze, elastic bandage, and surgical tape were applied to seal the wound. Upon regular inspection of the wounds, the bandages were changed^50^.

Stitches and bandages were removed on day 14, and the skin grafts were inspected on days 0, 1, 3, 5, 7, 10, 12, 14, and 21 after surgery. Two X-SCID rabbits were inspected up to day 56. Graft rejection was detected through the macroscopic appearance of graft necrosis, while graft necrosis was evaluated using a scale based on colour changes and scarring. At 21–56 days after transplantation, the animals were euthanised with pentobarbital. Graft skin tissues were fixed in 10% formalin solution, embedded in paraffin, and sectioned. The sections were stained with haematoxylin and eosin.

### Immunofluorescence staining

The grafted skin tissues were embedded in Tissue-Tek® Optimal Cutting Temperature compound (Sakura Fineteck, Tokyo, Japan) and sectioned at 10-μm thickness. The sections were washed thrice with Tris-buffered saline (TBS, TaKaRa Bio, Shiga, Japan) for 10 min and blocked for 1 h in TBS (TaKaRa Bio) containing 5% donkey serum (Jackson ImmunoResearch Laboratories, Inc., West Grove, PA, USA) and 0.3% Triton X-100 (FUJIFILM Holdings Corporation, Tokyo, Japan). The sections were then probed overnight at 4 °C with anti-CD3 (CD3-12; Abcam, Cambridge, UK, USA) and anti-CD45 (L12/201; Bio-Rad) antibodies in TBS containing 1% donkey serum and 0.3% Triton X-100. Subsequently, samples were again washed thrice with TBS for 5 min and were labelled for 1 hour at room temperature (20 °C–28 °C) with secondary antibodies conjugated with Alexa Flour 488 (Thermo Fisher Scientific). The sections were counterstained with Hoechst 33342 (Thermo Fisher Scientific) to visualise the nuclei and imaged using a fluorescence microscope (Axio Observer. D1, Carl Zeiss, Jena, Germany).

### Histochemical staining

Haematoxylin and eosin staining, tissues were fixed with 10% formaldehyde neutral buffered solution and embedded in paraffin and then 3–5-μm-thick sections were cut and stained with haematoxylin and eosin after deparaffinisation and hydration. The sections were imaged using a microscope equipped with a NanoZoomer-XR C12000 system (Hamamatsu Photonics, Hamamatsu, Japan) and an Axio Observer.D1 system. Grocott staining was performed by KAC Co., LTD. (Kyoto, Japan).

### Statistical analyses

All data are expressed as mean ± S.D. values. Statistical analyses were performed using the Welch’s t test. p values of <0.05 were considered to indicate statistical significance. All of statistical analyses were performed using the GraphPad Prism8 software (GraphPad Software Inc., San Diego, CA, USA).

## Supplementary information

Supplementary dataset 1.

## References

[1] Flanagan SP (1966). ‘Nude’, a new hairless gene with pleiotropic effects in the mouse. Genet. Res..

[2] Szadvari I, Krizanova O, Babula P (2016). Athymic nude mice as an experimental model for cancer treatment. Physiol. Res..

[3] Bosma GC, Custer RP, Bosma MJ (1983). A severe combined immunodeficiency mutation in the mouse. Nature.

[4] McCune J, Namikawa R, Shih C, Rabin L, Kaneshima H (1990). Pseudotypes in HIV-infected mice. Science (80-.)..

[5] Ohbo, B. K. *et al.* Modulation of hematopoiesis in mice with a truncated mutant of the interleukin-2 receptor gamma chain. *Blood* **87**, 956-967 (1996) 8562967

[6] Allenspach, E., Rawlings, D. J. & Scharenberg, A. M. *X-Linked Severe Combined Immunodeficiency. GeneReviews*® (1993). 20301584

[7] Mashimo, T. *et al*. Generation of Knockout Rats with X-Linked Severe Combined Immunodeficiency (X-SCID) Using Zinc-Finger Nucleases. *PLoS ONE* **5**, (2010) 10.1371/journal.pone.0008870 PMC2810328 20111598

[8] Sato, K. *et al.* Generation of a Nonhuman Primate Model of Severe Combined Immunodeficiency Using Highly Efficient Genome Editing. *Cell Stem Cell* **19**, 127-138 (2016) 10.1016/j.stem.2016.06.003 27374787

[9] Suzuki, S. *et al.* Il2rg Gene-Targeted Severe Combined Immunodeficiency Pigs. *Cell Stem Cell* **10**, 753-758 (2012) 10.1016/j.stem.2012.04.021 22704516

[10] Felsburg, P. J., Somberg, R. L., Hartnett, B. J., Henthorn, P. S. & Carding, S. R. Canine X-linked severe combined immunodeficiency. A model for investigating the requirement for the common gamma chain (gamma c) in human lymphocyte development and function. *Immunol. Res*. **17**, 63–73 (1998). 10.1007/BF02786431 9479568

[11] Graur, D., Duret, L. & Gouyt, M. Phylogenetic position of the order Lagomorpha (rabbits, hares and allies). *Nature* **379**, 333–335 (1996). 10.1038/379333a0 8552186

[12] Peng, X., Knouse, J. A. & Hernon, K. M. Rabbit Models for Studying Human Infectious Diseases. *Comp. Med*. **65**, 499–507 (2015). PMC4681244 26678367

[13] Tomozawa, Y. *et al*. Anti-tumor Effects of Sorafenib Administered at Different Time Points in Combination with Transarterial Embolization in a Rabbit VX2 Liver Tumor Model. *Cardiovasc. Intervent. Radiol.* **40**, 1763–1768 (2017). 10.1007/s00270-017-1719-9 28593395

[14] Panfil, A. R., Al-saleem, J. J. & Green, P. L. Virology: Research and Treatment. *Virol. (Auckl).* 49–59 10.4137/VRT.S12140.TYPE (2013). 10.4137/VRT.S12140 PMC4222344 25512694

[15] Ghivizzani, S. C. *et al*. Direct adenovirus-mediated gene transfer of interleukin 1 and tumor necrosis factor soluble receptors to rabbit knees with experimental arthritis has local and distal anti-arthritic effects. *Proc. Natl. Acad. Sci.* **95**, 4613–4618 (2002). 10.1073/pnas.95.8.4613 PMC22538 9539786

[16] Wu, Y., Zhang, C., Wu, J., Han, Y. & Wu, C. Angiogenesis and bone regeneration by mesenchymal stem cell transplantation with danshen in a rabbit model of avascular necrotic femoral head. *Exp. Ther. Med*. 163–171 10.3892/etm.2019.7556 (2019). 10.3892/etm.2019.7556 PMC6566092 31258650

[17] Klokkevold, P. R., Nishimura, R. D., Adachi, M. & Caputo, A. Osseointegration enhanced by chemical etching of the titanium surface. A torque removal study in the rabbit. *Clin. Oral Implants Res.* **8**, 442–7 (1997). 10.1034/j.1600-0501.1997.080601.x 9555202

[18] Hayashi, R. *et al.* Co-ordinated ocular development from human iPS cells and recovery of corneal function. *Nature* **531**, 376-380 (2016) 10.1038/nature17000 26958835

[19] Taylor, W. R. & Vaney, D. I. Diverse Synaptic Mechanisms Generate Direction Selectivity in the Rabbit Retina. *J. Neurosci.* **22**, 7712–7720 (2018). 10.1523/JNEUROSCI.22-17-07712.2002 PMC6757986 12196594

[20] Ytrehus, K., Liu, Y. & Downey, J. M. Preconditioning protects ischemic rabbit heart by protein kinase C activation. *Am. J. Physiol. Circ. Physiol.* **266**, H1145–H1152 (2017). 10.1152/ajpheart.1994.266.3.H1145 8160817

[21] Peuster, M. A novel approach to temporary stenting: degradable cardiovascular stents produced from corrodible metal---results 6-18 months after implantation into New Zealand white rabbits.* Heart* **86**, 563–569 (2001). 10.1136/heart.86.5.563 PMC1729971 11602554

[22] Buja, L. M., Kita, T., Goldstein, J. L., Watanabe, Y. & Brown, M. S. Cellular pathology of progressive atherosclerosis in the WHHL rabbit. An animal model of familial hypercholesterolemia. *Arterioscler. An Off. J. Am. Hear. Assoc. Inc.* **3**, 87–101 (2011). 10.1161/01.atv.3.1.87 6824499

[23] Fan, J. *et al*. Rabbit models for the study of human atherosclerosis: From pathophysiological mechanisms to translational medicine. *Pharmacol. Ther*. **146**, 104–119 (2015). 10.1016/j.pharmthera.2014.09.009 PMC4304984 25277507

[24] Fan, J. *et al*. Principles and Applications of Rabbit Models for Atherosclerosis Research. *J. Atheroscler. Thromb.* **25**, 213–220 (2018). 10.5551/jat.RV17018 PMC5868506 29046488

[25] Fan, J. & Watanabe, T. Transgenic rabbits as therapeutic protein bioreactors and human disease models. *Pharmacol. Ther.* **99**, 261–282 (2003). 10.1016/s0163-7258(03)00069-x 12951161

[26] Spieker-Polet, H., Sethupathi, P., Yam, P. C. & Knight, K. L. Rabbit monoclonal antibodies: generating a fusion partner to produce rabbit-rabbit hybridomas. *Proc. Natl. Acad. Sci.* **92**, 9348–9352 (2006). 10.1073/pnas.92.20.9348 PMC40982 7568130

[27] Sui, T. *et al.* CRISPR/Cas9-mediated mutation of PHEX in rabbit recapitulates human X-linked hypophosphatemia (XLH). *Hum. Mol. Genet.* **25**, 2661–2671 (2016). 10.1093/hmg/ddw125 27126636

[28] Yang, D. *et al*. Effective gene targeting in rabbits using RNA-guided Cas9 nucleases. *J. Mol. Cell Biol*. **6**, 97–99 (2014). 10.1093/jmcb/mjt047 PMC3983410 24403564

[29] HONDA, A. *et al*. Single-step generation of rabbits carrying a targeted allele of the tyrosinase gene using CRISPR/Cas9.* Exp. Anim.* **64**, 31–37 (2014). 10.1538/expanim.14-0034 PMC4329513 25195632

[30] Yan, Q. *et al*. Generation of multi-gene knockout rabbits using the Cas9/gRNA system. *Cell Regeneration.* **3**, 3:12 (2014) 10.1186/2045-9769-3-12 PMC4230364 25408890

[31] Song, J. *et al.* Production of immunodeficient rabbits by multiplex embryo transfer and multiplex gene targeting. *Sci. Rep.* **7**, 1–9 (2017). 10.1038/s41598-017-12201-0 PMC5610260 28939872

[32] Puck, J. M., Nussbaum, R. L. & Conley, M. E. Carrier detection in X-linked severe combined immunodeficiency based on patterns of X chromosome inactivation. *J. Clin. Invest*. **79**, 1395–1400 (1987). 10.1172/JCI112967 PMC424401 2883199

[33] Niu, Y. *et al.* Generation of Gene-Modified Cynomolgus Monkey via Cas9/RNA-Mediated Gene Targeting in One-Cell Embryos. *Cell* **156**, 836-843 (2014) 10.1016/j.cell.2014.01.027 24486104

[34] Shin, H. Y.* et al*. CRISPR/Cas9 targeting events cause complex deletions and insertions at 17 sites in the mouse genome. *Nat. Commun.* **8**, 1–10 (2017). 10.1038/ncomms15464 PMC5460021 28561021

[35] Cao, X. *et al.* Defective lymphoid development in mice lacking expression of the common cytokine receptor γ chain.* Immunity* **2**, 223-238 (1995) 10.1016/1074-7613(95)90047-0 7697543

[36] Wang MM. *et al*. Induction of immune tolerance and altered cytokine expression in skin transplantation recipients. *Kaohsiung J Med Sci*. **34**, 330–334 (2018). 10.1016/j.kjms.2018.01.005 PMC11915584 29747776

[37] Samstein B. *et al*. Toll-like receptor-4 and allograft responses. *Transplantation* **168**, 628–632 (2003). 10.1097/01.TP.0000110792.38434.F4 14966433

[38] Zhou, L., Xiao, Q., Bi, J., Wang, Z. & Li, Y. RabGTD: A comprehensive database of rabbit genome and transcriptome. *Database* **2018**, 1–8 (2018). 10.1093/database/bay075 PMC6047408 30010730

[39] Klempnauer J1, Steiniger B, Marquarding E, Vogt P, Lipecz A, Wonigeit K, G. E. Effects of the RT1.C Region in Rat Allotransplantation. 713–5 doi:3274851 (1987). 3274851

[40] C. K. CHAI. Effects of Inbreeding in Rabbits. *Journal of Heredity* **60**, 64-70, (1969) 10.1093/oxfordjournals.jhered.a107934 5800994

[41] Rigatti, L. H., Toptan, T., Newsome, J. T., Moore, P. S. & Chang, Y. Identification and Characterization of Novel Rat Polyomavirus 2 in a Colony of X-SCID Rats by P-PIT assay. *mSphere* **1**, 1–13 (2016). 10.1128/mSphere.00334-16 PMC5177731 28028546

[42] Song, J. *et al*. Bacterial and pneumocystis infections in the lungs of gene-knockout rabbits with severe combined immunodeficiency. *Front. Immunol*. **9**, 1–9 (2018). 10.3389/fimmu.2018.00429 PMC5854650 29593714

[43] Lavoie, M. C. *et al*. Pneumocystis carinii infection in transgenic B cell-deficient mice. *J. Infect. Dis*.** 173**, 1034–1037 (1996). 10.1093/infdis/173.4.1034 8603947

[44] Sanchez, C. A. *et al*. Exploring transplacental transmission of Pneumocystis oryctolagi in first-time pregnant and multiparous rabbit does. *Med. Mycol*. **45**, 701–707 (2007). 10.1080/13693780701531156 18027254

[45] Cere, N., Polack, B. & Coudert, P. Obtaining a Pneumocystis-free rabbit breeding stock (Oryctolagus cuniculus). *J. Eukaryot. Microbiol*. **44**, 19S-20S (1997). 10.1111/j.1550-7408.1997.tb05747.x 9508411

[46] Samata, B. *et al*. X-linked severe combined immunodeficiency (X-SCID) rats for xeno-transplantation and behavioral evaluation. *J. Neurosci. Methods* **243**, 68–77 (2015). 10.1016/j.jneumeth.2015.01.027 25662444

[47] Yoshimi, K., Kaneko, T., Voigt, B. & Mashimo, T. Allele-specific genome editing and correction of disease-associated phenotypes in rats using the CRISPR-Cas platform. *Nat. Commun.* **5**, 1–9 (2014). 10.1038/ncomms5240 PMC4083438 24967838

[48] Yoshimi, K. *et al*. SsODN-mediated knock-in with CRISPR-Cas for large genomic regions in zygotes. *Nat. Commun.* **7**, 1–10 (2016). 10.1038/ncomms10431 PMC4736110 26786405

[49] Sobajima, S. *et al*. Quantitative analysis of gene expression in a rabbit model of intervertebral disc degeneration by real-time polymerase chain reaction. *Spine J.* **5**, 14–23 (2005). 10.1016/j.spinee.2004.05.251 15653081

[50] Ding, J. & Tredget, E. E. Transplanting Human Skin Grafts onto Nude Mice to Model Skin Scars. *In Methods Mol. Biol*. 65–80. 10.1007/978-1-4939-7113-8_5 (2017). 10.1007/978-1-4939-7113-8_5 28836195

