# Supplementary material for: Generation of knockout rabbits with X-linked severe combined immunodeficiency (X-SCID) using CRISPR/Cas9
Source: Sci Rep. 2020 Jun 19;10:9957. doi: 10.1038/s41598-020-66780-6 (PMC7305219; doi:10.1038/s41598-020-66780-6)

Generation of knockout rabbits with X-linked severe combined immunodeficiency (X-SCID) using CRISPR/Cas9

Yoshiko Hashikawa[1, 2, 3], Ryuhei Hayashi[1, 3*], Masaru Tajima[2, 4], Toru Okubo[3], Shohei Azuma[1], Mitsuru Kuwamura[5], Naofumi Takai[6], Yasuyuki Osada[6], Yayoi Kunihiro[4], Tomoji Mashimo[4, 7*], Kohji Nishida[1, 8]

[1] Department of Ophthalmology, Osaka University Graduate School of Medicine, Osaka, Japan

[2] Institute of Large Laboratory Animal Sciences, Osaka University Graduate School of Medicine, Osaka, Japan

[3] Department of Stem Cells and Applied Medicine, Osaka University Graduate School of Medicine, Osaka, Japan

[4] Institute of Experimental Animal Science, Graduate School of Medicine, Osaka University, Osaka, Japan

[5] Osaka Prefecture University School of Life and Environmental Sciences Veterinary pathology, Osaka, Japan

[6] KITAYAMA LABES CO.,LTD., Nagano, Japan

[7] Laboratory Animal Research Center, Institute of Medical Science, The University of Tokyo, Tokyo, Japan

[8] Integrated Frontier Research for Medical Science Division, Institute for Open and Transdisciplinary Research Initiatives (OTRI), Osaka University, Osaka, Japan

Correspondence and requests for materials should be addressed to R.H. (email: ryuhei.hayashi@ophthal.med.osaka-u.ac.jp) or to T.M. (email: mashimo@ims.u-tokyo.ac.jp)

Supplementary Figure 1.

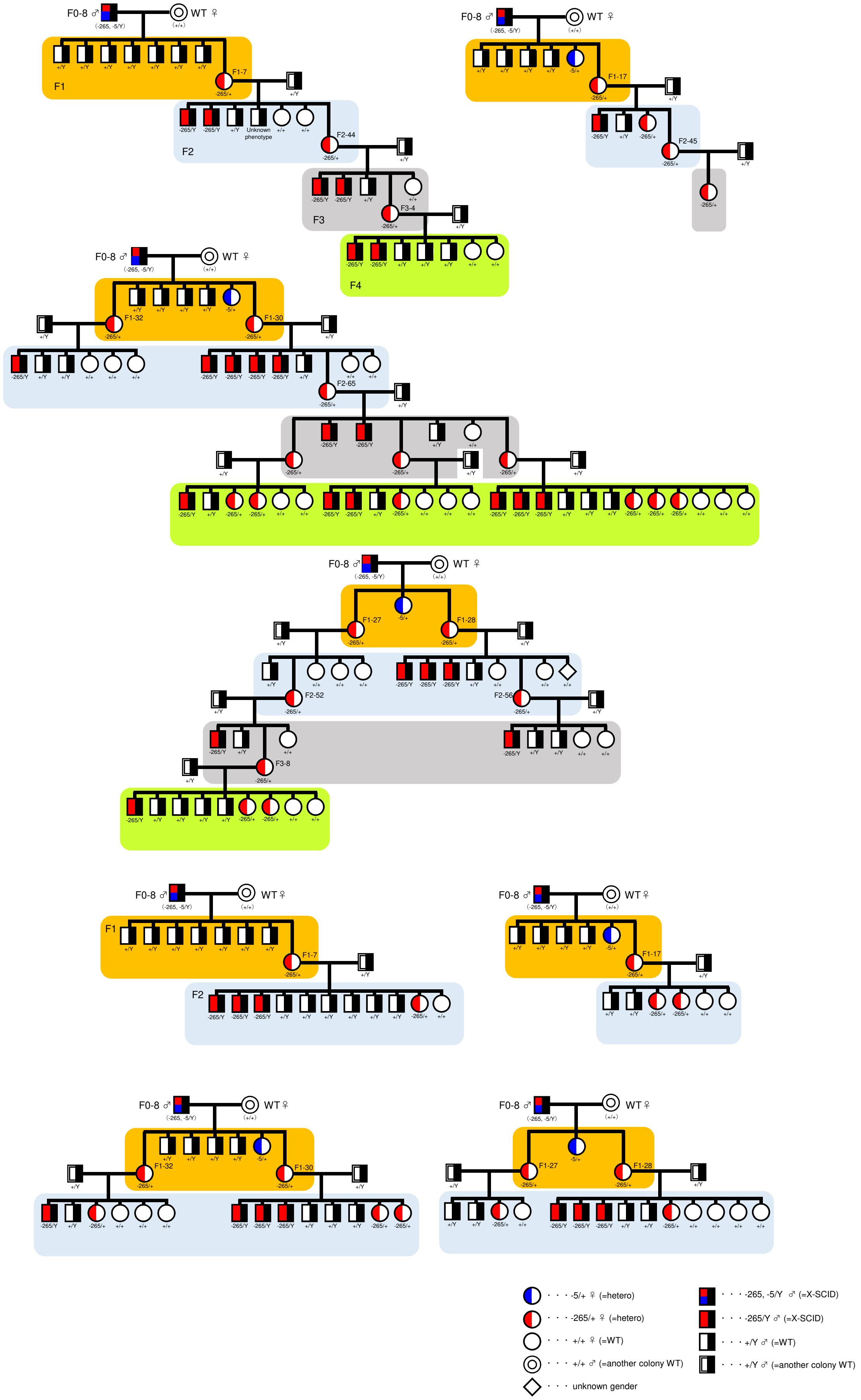

Supplementary Figure 2.

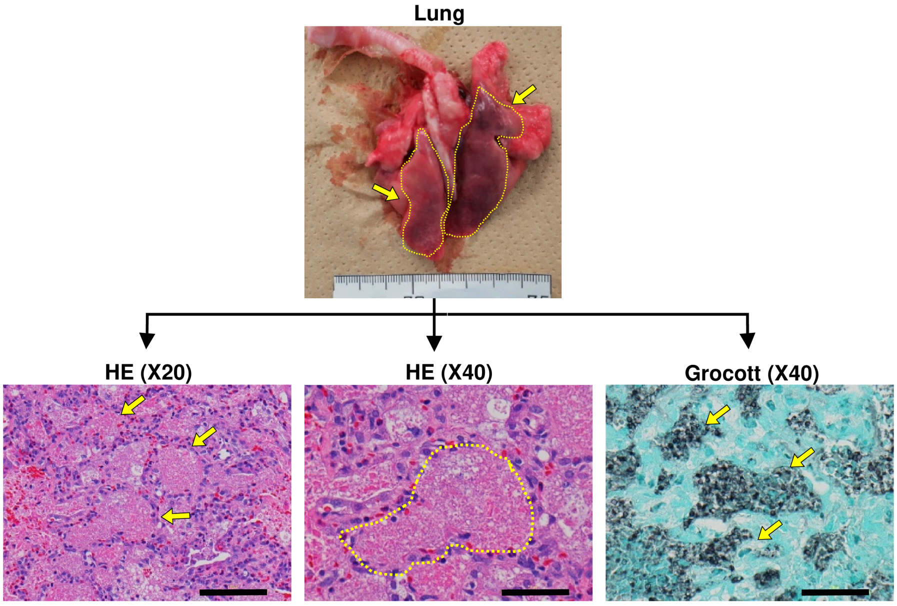

Supplementary Figure 3.

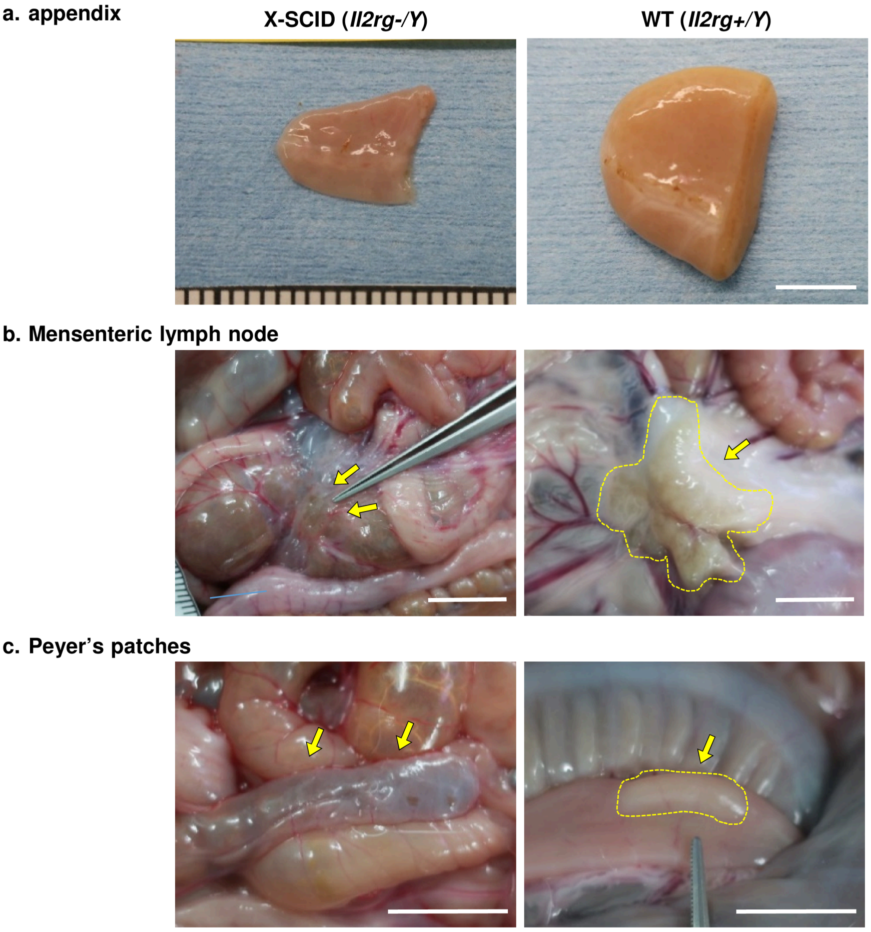

Supplementary Figure 4.

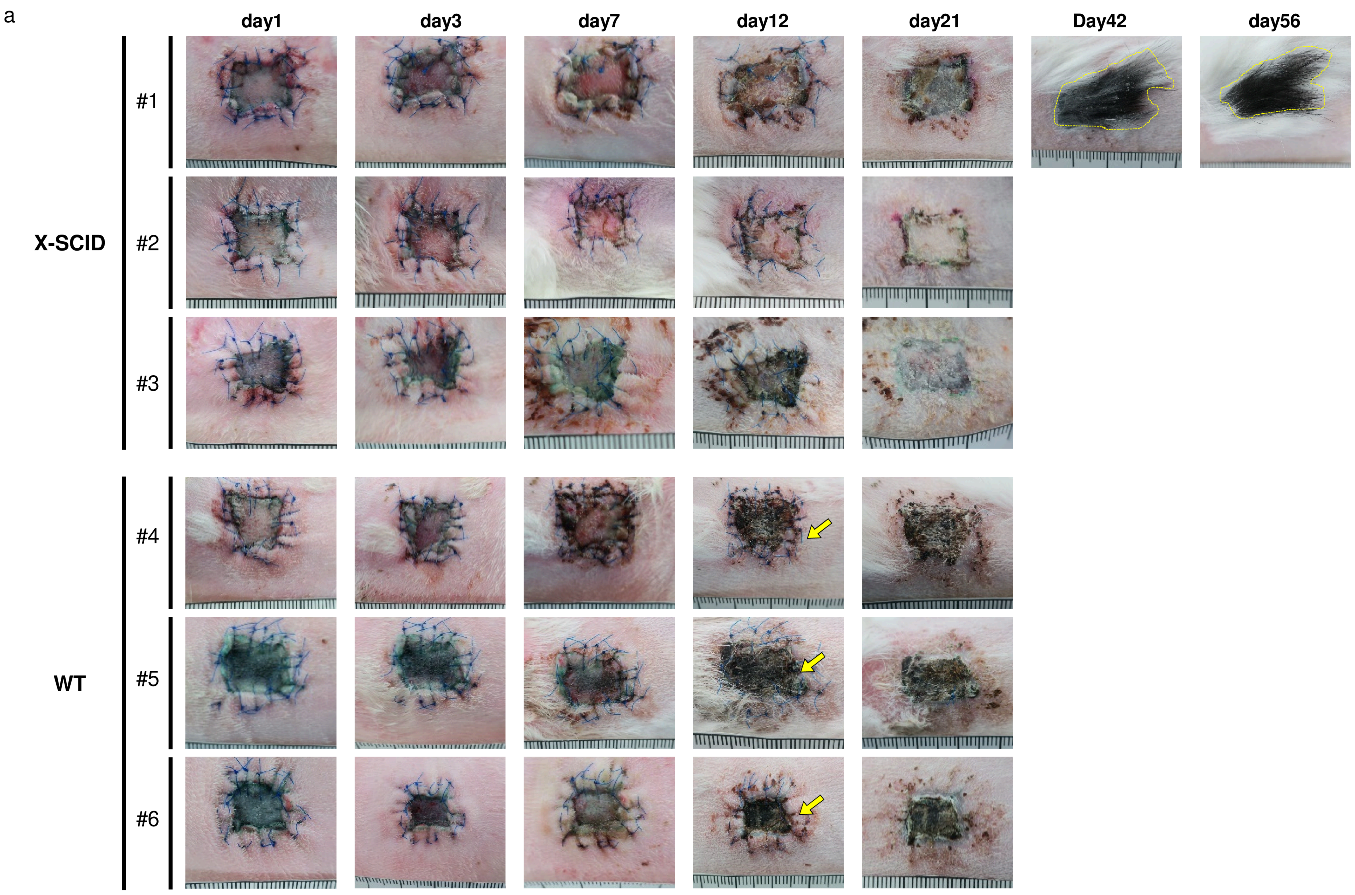

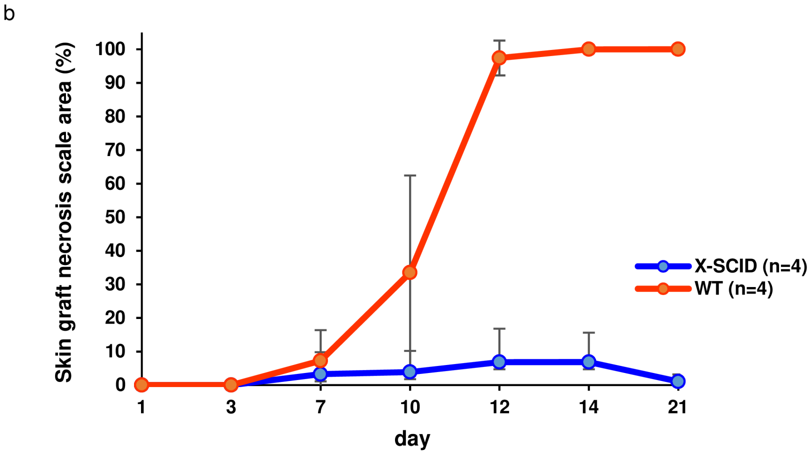

Supplementary Table 1.

parent			pups generation	*Il2rg* genotype				total
				male ♂		female ♀		
mutant		WT		X-SCID (%)	WT (%)	Hetero (%)	WT (%)	
♂ (-265, -5)	×	♀ (+/+)	F1	0 (0)	15 (50)	15 (50)	0 (0)	30
♀ (-265/+)	×	♂ (+/+)	F2	21 (26.3)	23 (28.8)	14 (17.5)	22 (27.5)	80
♀ (-265/+)	×	♂ (+/+)	F3	6 (27.3)	5 (22.7)	6 (27.3)	5 (22.7)	22
♀ (-265/+)	×	♂ (+/+)	F4	8 (19.0)	13 (31.0)	9 (21.4)	12 (28.6)	42

Supplementary Table 2.

	Spleen weight (g)
X-SCID (*Il2rg-/Y*)<br>n=4	1.2 ± 0.09
WT (*Il2rg+/Y*)<br>n=5	1.0 ± 0.09

Supplementary Table 3.

Gene	Sequence		GeneBank accesstion No.
Il2rg (gDNA)	Forward	AGGATCCTCACACCCAGTGA	XM-008272760
	Reverse	TAAGCTTCCTCGTGGTCTGC	
	Forward	CCTGGGTCCCTCAGTGTTT	XM-008272760
	Reverse	TGTCTCCATGGGTTAGGTCG *	
Il2rg (mRNA)	Forward	GAAGAGGAAGCGCCATGTTG	XM-008272760
	Reverse	TTAAGCTTCCTCGTGGTCTGC	
GAPDH	Forward	GGAGAAAGCTGCTAA	L23961
	Reverse	ACGACCTGGTCCTCGGTGTA	

* The primer was designed based on our sequencing results of Japanese White rabbit.

Supplementary Figure and Table Legends

Supplementary Figure 1. Pedigrees generated from sequence analyses of the F0 founder, F1, F2, F3, and F4 progeny.

Supplementary Figure 2. X-SCID rabbit abnormality lung.

Macroscopic and Histological analysis of the lung abnormality area (yellow circled area) of X-SCID rabbit (25 weeks old, male) stained with H&E and Grocott. HE (X20) scale bar is 100μm, HE (X40) and Grocott (X40) scale bar is 50μm.

Supplementary Figure 3. Phenotype during organogenesis in X-SCID rabbits.

(a) Histological analysis of the appendix of X-SCID and WT rabbits (25 weeks old, male). Scale bar, 5 mm. **(b)** Histological analysis of the mesenteric lymph node (yellow circled area) of X-SCID and WT rabbits (25 weeks old, male). Scale bar, 10 mm. **(c)** Histological analysis of the Peyer's patches (yellow circled area) of X-SCID and WT rabbits (25 weeks old, male). Scale bar, 10 mm.

Supplementary Figure 4. Allogeneic skin transplantation.

(a) Morphological analysis of temporal changes in the skin graft. The area demarcated by the yellow circled area shows black hair, characteristic of Dutch rabbits. The yellow arrow indicates darkening of the transplanted skin. **(b)** Temporal changes in the area of skin graft necrosis.

Supplementary Table 1. Number of offspring from F1 to F4 generations in the *Il2rg* genotype.

Supplementary Table 2. Weight of spleen.

Supplementary Table 3. Primer sequences.

Supplementary Gel Images for

Generation of knockout rabbits with X-linked severe combined immunodeficiency (X-SCID) using CRISPR/Cas9

Yoshiko Hashikawa[1, 2, 3], Ryuhei Hayashi[1, 3*], Masaru Tajima[2, 4], Toru Okubo[3], Shohei Azuma[1], Mitsuru Kuwamura[5], Naofumi Takai[6], Yasuyuki Osada[6], Yayoi Kunihiro[4], Tomoji Mashimo[4, 7*], Kohji Nishida[1, 8]

[1] Department of Ophthalmology, Osaka University Graduate School of Medicine, Osaka, Japan

[2] Institute of Large Laboratory Animal Sciences, Osaka University Graduate School of Medicine, Osaka, Japan

[3] Department of Stem Cells and Applied Medicine, Osaka University Graduate School of Medicine, Osaka, Japan

[4] Institute of Experimental Animal Science, Graduate School of Medicine, Osaka University, Osaka, Japan

[5] Osaka Prefecture University School of Life and Environmental Sciences Veterinary pathology, Osaka, Japan

[6] KITAYAMA LABES CO.,LTD., Nagano, Japan

[7] Laboratory Animal Research Center, Institute of Medical Science, The University of Tokyo, Tokyo, Japan

[8] Integrated Frontier Research for Medical Science Division, Institute for Open and Transdisciplinary Research Initiatives (OTRI), Osaka University, Osaka, Japan

Correspondence and requests for materials should be addressed to R.H. (email: ryuhei.hayashi@ophthal.med.osaka-u.ac.jp) or to T.M. (email: mashimo@ims.u-tokyo.ac.jp)

Area of interest are highlighted by a white square.
Labels correspond to those in the main paper.

Figure 1a

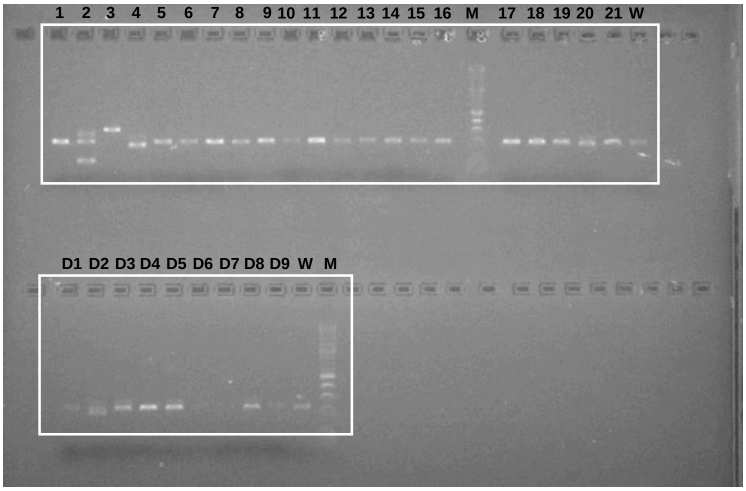

Figure 3c

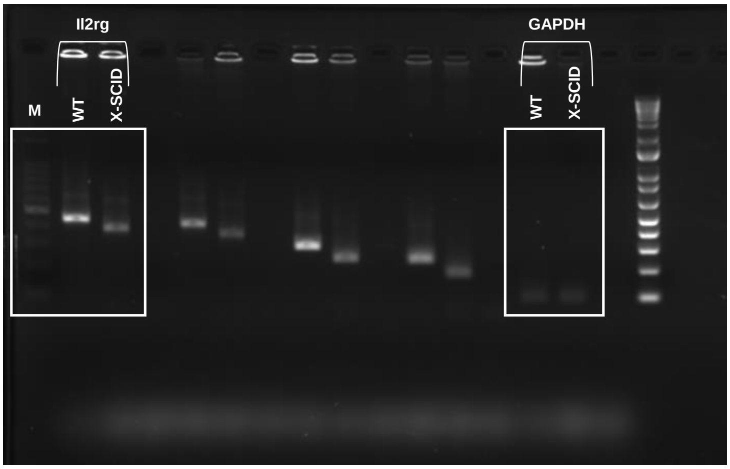

Supplement: Supplementary file 1 — Supplementary dataset 1. [file 41598_2020_66780_MOESM1_ESM.pdf]
